# Socioeconomic factors are associated with the prognosis of Thyroid Cancer

**DOI:** 10.7150/jca.52329

**Published:** 2021-03-05

**Authors:** Yu Li, Da Huang, Baoxin Wang, Wei Mao, Xinwei Chen, Pin Dong

**Affiliations:** 1 Department of Otolaryngology Head and Neck Surgery, Shanghai General Hospital, Shanghai Jiao Tong University School of Medicine, Shanghai 200080, China.; 2 Ruijin Clinical School, Shanghai Jiao Tong University School of Medicine, Shanghai 200023, China 200080.

**Keywords:** socioeconomic factors, thyroid cancer, marital status, Yost index, SEER

## Abstract

**Background:** Thyroid adenomas/adenocarcinomas are the most common type of thyroid cancer. The impact of socioeconomic factors on the prognosis of thyroid cancer is unclear.

**Methods:** Clinical information and socioeconomic factors were obtained from the Surveillance, Epidemiology, and End Results Database (SEER) 18 Registries Custom Database. The association between thyroid adenomas/adenocarcinomas and socioeconomic factors including gender, race/ethnicity, insurance status, marital status, living area, and Yost index (including education, income, working, etc.) were fully evaluated.

**Results:** A total of 136,313 patients between 2010 and 2016 were finally included in the present study. Among them, 126,160 patients were diagnosed with the single malignancy. Median follow-up time was 64 months. In general, non-Hispanic Asian or Pacific Islander and Hispanic patients had significantly better survival than non-Hispanic White patients (All *P* <0.05). Patients insured by Medicaid had significantly poorer cancer-specific survival (CSS, hazard ratio, HR=2.15, *P* <0.001) and overall survival (OS, HR=2.42, P <0.001) than those insured by commercial insurance or Medicare. In addition, divorced or widowed status, rural living location and low Yost index were significantly associated with poor CSS and OS of thyroid adenomas/adenocarcinomas (All *P* <0.05). Subgroup analyses showed similar results in patients who received surgical procedure, as well as in patients who received both surgical and radiation therapy. Multivariate analyses suggested that insurance status, marital status and Yost index remained significantly associated with CSS and OS (all *P* <0.05).

**Conclusions:** Socioeconomic factors, including insurance status, marital status, living area, and Yost index, were significant predictors for the survival of thyroid adenomas/adenocarcinomas.

## Introduction

With the estimated 567,233 new cases and 41,071 deaths worldwide, thyroid cancer has become the ninth most common malignant tumor, especially in highly developed regions such as Northern America, Australia/New Zealand, South Korea, etc. 1. In the United States, the incidence rate in women is 3 times higher than in men, which ranked in fifth place among women population 2. As a type of relatively indolent malignancy, thyroid cancer has a mortality rate of 0.5 per 100,000 men and women per year 3. Previous studies demonstrated that multiple socioeconomic factors might influence the treatment selection and further prognosis of thyroid cancer, such as insurance status, marital status, living area, etc. 4-12. However, all the above studies focused on either a sole socioeconomic factor or a sole histology subtype. There was still not a comprehensive study about the effect of the socioeconomic factors on thyroid cancer's prognosis.

In this study based on the Surveillance, Epidemiology, and End Results (SEER) Custom Database, we aimed to further examine whether socioeconomic factors influenced the survival outcomes of patients with thyroid cancer, after adjusting the baseline clinical characteristics including race/ethnicity, gender, and stage.

## Materials and methods

### Study population

The study population of the current study was included from the SEER 18 Registries Custom Database linked to Time-Dependent Census-Tract Attributes between 2010 and 2016. The inclusion criteria were: (a) patients diagnosed with thyroid adenomas/adenocarcinomas with pathological confirmation (International Classification of Disease for Oncology [ICD-O-3] code 8140-8389); (b) patients with follow-up information and at least one record of socioeconomic factors including gender, race/ethnicity, insurance status, marital status, living area, and Yost index. Yost index was derived from median household income, median house value, median rent, percent below 150% of the poverty line, education index, percent working class, and percent unemployed 13. Patients with missing follow-up information or socioeconomic information were excluded from the current study. Treatment information including surgical treatment and radiation therapy was also obtained for subgroup analyses of treatment effects. All the analyses were performed in patients with primary thyroid adenomas/adenocarcinomas (who were registered as “One Primary Only” in SEER database) and the entire population (regardless of whether the thyroid cancer was the only primary tumor the patient had).

The study was approved by the Institutional Review Board of Shanghai General Hospital, Shanghai, China.

### Statistical analysis

Descriptive analyses were performed to show the baseline characteristics of the study population. Chi-square test was used to evaluate the association between 5-year or 10-year survival and different factors. Univariate Cox regression was used for the survival analysis of different factors. Multivariate Cox regression analyses were also applied to evaluate the independent hazard ratio (HR) of different factors adjusting for gender, race/ethnicity, insurance status, marital status, living area (two-category Rural Urban Commuting Area [RUCA] codes), Yost index tertiles, and SEER Summary Stage 14. All statistical analyses were performed with R software (Version 4.0.0) 15. A two-sided *P*-value <0.05 was considered statistically significant.

## Results

A total of 136,313 patients between 2010 and 2016 were finally included in the present study. Among them, 126,160 patients were diagnosed with the single malignancy (primary thyroid adenomas/adenocarcinomas only). Baseline characteristics of patients with primary thyroid cancer are shown in **Table 1**. Median follow-up time was 64 months in patients with the single malignancy.

The association between different socioeconomic factors and 5-year or 10-year survival is also shown in**Table S1** and** Table 1,** respectively. Male patients had ~2.8-fold increased risk of 5-year or 10-year cancer-specific deaths and ~2.5-fold increased all-cause deaths than females (all *P* <0.05). Compared to non-Hispanic White patients, non-Hispanic Black patients had a significantly higher risk of all-cause deaths (5-year odds ratio, OR=1.40, 95% confidence interval, 95%CI: 1.24-1.57; 10-year OR=1.34, 95%CI: 1.22-1.47, both *P* <0.001, **Table S1**); however, non-Hispanic Black patients had less 5-year cancer-specific deaths (OR=0.62, 95%CI: 0.51-0.74, *P* <0.001, **Table S1**). In univariate Cox regression survival analysis, no significant differences in cancer-specific survival (CSS) were observed between non-Hispanic Black and White patients (*P*=0.40, **Table 2**); however, non-Hispanic Black patients had poorer CSS and overall survival (OS) than non-Hispanic White patients among those who receive surgical treatment (HR_CSS_=1.32, HR_OS_=1.55, *P* <0.001, **Table 2** and**Table S2**). Similarly, non-Hispanic Asian or Pacific Islander (HR=1.28, 95%CI: 1.20-1.36, *P* <0.001) and Hispanic patients (HR=1.10, 95%CI: 1.07-1.14, *P* <0.001, **Table 2**) had poorer CSS than non-Hispanic White patients in patients undertaking surgical treatment. In patients received both surgical treatment and radiation therapy, non-Hispanic Asian or Pacific Islander patients had significantly poorer CSS than non-Hispanic White patients (HR=1.11, 95%CI: 1.03-1.18, *P*=0.003), while Hispanic patients had similar CSS with non-Hispanic White patients (HR=1.03, 95%CI: 1.00-1.06, *P*=0.09, **Table S3**). Multivariate Cox regression analysis suggested different results (**Table 3**). After adjusting for multiple variables including gender, insurance status, marital status, residency location, Yost index and tumor stages, the results suggested that both non-Hispanic Asian or Pacific Islander patients and Hispanic patients had significantly better CSS and OS than non-Hispanic White patients. Similar results were also observed in subgroups with definitive treatment (**Table S2** and**Table S3**).

Patients insured by Medicaid would have ~2-fold increased risk of 5-year/10-year cancer-specific deaths and all-cause deaths (all *P* <0.05, **Table S1** and** Table 1**). These patients also had significantly poorer CSS (HR=2.99 in patients received surgical treatment, HR=2.28 in patients received both surgical treatment and radiation therapy, both *P* <0.05, **Table 2** and**Table S3**) and OS (HR=2.94 in patients received surgical treatment, HR=3.02 in patients received both surgical treatment and radiation therapy, both *P* <0.05, **Table S2** and**Table S3**). Multivariate cox regression analysis suggested that insurance status of Medicaid was a significantly independent factor for poorer disease survival (**Table 3, Table S2** and **Table S3**).

Divorced status and widowed status were significantly associated with increased risk of 5-year and 10-year cancer-specific deaths and all-cause deaths (all *P* <0.05, **Table S1 and Table 1**). In patients who received surgical treatment, divorced patients and widowed patients would have significantly poorer CSS than married patients, with HR=1.35 (95%CI: 1.26-1.46, *P* <0.001) and HR=1.92 (95%CI: 1.85-2.00,* P* <0.001, **Table 2**), respectively. Similar results were observed in patients undertaking both surgical and radiation therapy (**Table S3**). In addition, marital status was also associated with OS of the disease (**Table S2**). When adjusting for other factors, the association between marital status and CSS/OS remained significant (**Table 3, Table S2** and **Table S3**).

In univariate analysis, the rural living location was significantly associated with poorer prognosis of the disease (**Table 2**). However, it was not an independent predictor for cancer prognosis after multivariate adjustment (**Table 3**).

We then investigated whether Yost index would affect the survival of thyroid adenomas/adenocarcinomas. Results suggested that patients with a higher Yost index would have significantly better survival than patients with a low Yost index (**Table 2**). The association between Yost index and CSS/OS remained significant after adjusting for different factors in multivariate analysis, especially in patients with the highest Yost index (for CSS, HR=0.77 in patients received surgical treatment, HR=0.76 in patients received both surgical treatment and radiation therapy, both *P* <0.05; for OS, HR=0.60 in patients received surgical treatment, HR=0.62 in patients received both surgical treatment and radiation therapy, both *P* <0.001,** Table 3, Table S2 and Table S3**). In patients with a medium Yost index, similar trends could be observed though not all the associations reached statistical significance level.

Similar analyses were performed in all the patients who diagnosed with thyroid adenomas/adenocarcinomas (could be diagnosed with multiple cancers), and the results were shown in **Table S4** and **Table S5**. We observed similar associations.

## Discussion

In order to better understand the influence of socioeconomic factors on disease survival of thyroid cancer, we conducted the present study using the largest database of SEER with follow-up information. We found that socioeconomic factors were significantly associated with CSS and OS of thyroid adenomas/adenocarcinomas. In particular, male gender, insured by Medicaid, divorced or widowed marital status, and low Yost index (including education, income, working, and housing situation, etc.) were independent and significant risk factors of poorer CSS. In addition to these factors, certain races (non-Hispanic Black patients had poorer OS, while Hispanic patients had significantly better OS) were significantly associated with OS of the disease.

Brown *et al.* demonstrated that black patients had similar 5-year survival rates (*P* =0.273) as white patients in an equal access healthcare system in the United States 16. However, similar analyses showed significant difference in the present study using the SEER database. Non-Hispanic Black patients had a significantly higher risk of all-cause deaths compared to non-Hispanic White patients, although multivariate analysis did not observe significant difference in either CSS or OS between non-Hispanic Black and White patients.

Several retrospective studies demonstrated that married patients with thyroid cancer had survival advantages in the United States, which was confirmed by our integrated analyses 4, 8. Shi *et al* reported widowed and divorced patients with differentiated thyroid cancer would have significantly higher cancer mortality with an HR of 2.95 in widowed patients and an HR of 1.78 in divorced patients 9. Similar results were also found by Merrill *et al* when models were adjusted for clinical variables 10. In our study, we evaluated the effect of marital statuses in patients with adenomas/adenocarcinomas regardless of histology subtypes. We also adjusted for the other socioeconomic factors in our analyses. Our results demonstrated that marital status could be a strong predictor for prognosis of thyroid adenomas/adenocarcinomas. An interesting finding was that even though unmarried status included single, divorced, widowed, and separated sub-status, their impacts on the prognosis were different. In the current study, patients who were single would have significantly better CSS and OS. And a separated marital status did not associate with the disease prognosis. Such pheromone was worth further investigation incorporating social connection, psychological factors and mental health status.

County poverty level (low versus high) was used to determine socioeconomic status in the previous studies 8, 12, 17, 18. High poverty level was significantly associated with both poor CSS and OS in patients with well-differentiated 18 and anaplastic thyroid cancer 12, 17. However, no significant survival difference across poverty level was found in patients with medullary thyroid cancer 8. To the best of our knowledge, this is the first study to evaluate the impact of Yost index on prognosis of thyroid adenomas/adenocarcinomas. We found that patients with high tertile of Yost index had significantly better OS and CSS than those with low tertile, even after adjusting for clinical variables in our multivariate models. Such results indicated that Yost index was an independent predictor for thyroid cancer.

A recent study by McDow *et al* indicated that urban patients with thyroid cancer would have better long-term survival compared to rural patients from 2000 to 2012 19. Similar results were found in our univariate analyses; however, there was no significant association between RUCA category and survival outcomes in our multivariate models based on data from 2010 to 2016. This might be because that residency location would be highly associated with other socioeconomic factors such as housing, income, working status, etc. The effect of the residency location could be represented by Yost Index in the current study, and thus became insignificant in multivariate analysis. Residency location is an important indicator for the accessibility to medical resources in the United States; however, a binary classification of urban vs. rural may not be appropriate: (1) rural areas near metropolitan district may have significantly better accessibility to medical resources than those in, for example, mountain areas; (2) sub-urban areas with the vast majority of middle-class households are hardly to be classified to this binary system. Therefore, rather than using residency location as an indicator for cancer prognosis, more comprehensive factors such as Yost index may be more applicable.

A notable limitation was that we did not perform additional subgroup analyses for patients with different subtypes of histology. In the current study, our purpose was to investigate the association between the socioeconomic factors and the survival of all the adenomas/adenocarcinomas in thyroid gland. The potential heterogeneities among different histology subtypes should be investigated in the future studies.

## Conclusions

Socioeconomic factors, including insurance status, marital status, living area, and Yost index, were significant predictors for the survival of thyroid adenomas/adenocarcinomas.

## Supplementary Material

Supplementary tables.

## Figures and Tables

**Table 1:** Comparison of 10-year cancer-specific survival outcomes by different factors in patients with primary thyroid cancer^ a^

Characteristics	Primary Thyroid Cancer (N = 126,160)	10-year CSS (N of Events = 2,253)		
		n of Events (%)	OR (95%CI)	*P*
**Gender**				
Female	98,293 (77.9)	1,264 (1.3)	1.00 (Ref.)	/
Male	27,867 (22.1)	989 (3.5)	2.82 (2.59-3.08)	<0.001
**Race/Ethnicity**				
NH White	81,970 (65.0)	1,441 (1.8)	1.00 (Ref.)	/
NH Black	8,480 (6.7)	151 (1.8)	1.01 (0.85-1.20)	0.91
NH API	13,357 (10.6)	288 (2.2)	1.23 (1.08-1.40)	0.002
NH AI/AN	569 (0.5)	13 (2.3)	1.31 (0.69-2.26)	0.43
Hispanic	20,331 (16.1)	358 (1.8)	1.00 (0.89-1.13)	1.00
NH Unknown	1,453 (1.2)	2 (0.1)	/	/
**Insurance Status**				
Insured	79,429 (63.0)	867 (1.1)	1.00 (Ref.)	/
Medicaid	9,542 (7.6)	192 (2.0)	1.86 (1.58-2.18)	<0.001
Uninsured	2,448 (1.9)	34 (1.4)	1.28 (0.88-1.80)	0.20
Unknown	34,741 (27.5)	1160 (3.3)	/	/
**Marital Status**				
Married	76,770 (60.9)	1,177 (1.5)	1.00 (Ref.)	/
Single	27,305 (21.6)	320 (1.2)	0.76 (0.67-0.86)	<0.001
Divorced	8,417 (6.7)	189 (2.2)	1.48 (1.26-1.72)	<0.001
Widowed	5,394 (4.3)	452 (8.4)	5.87 (5.24-6.58)	<0.001
Separated	1,079 (0.9)	13 (1.2)	0.78 (0.41-1.35)	0.45
Unmarried^ b^	218 (0.2)	0 (0.0)	/	/
Unknown	6,977 (5.5)	102 (1.5)	/	/
**RUCA Category**				
Urban	112,655 (89.3)	1,926 (1.7)	1.00 (Ref.)	/
Rural	7,983 (6.3)	207 (2.6)	1.53 (1.32-1.77)	<0.001
Unknown	5,522 (4.4)	120 (2.2)		
**Yost tertile**				
Low	28,697 (22.7)	628 (2.2)	1.00 (Ref.)	/
Middle	39,569 (31.4)	754 (1.9)	0.87 (0.78-0.97)	0.01
High	50,381 (39.9)	726 (1.4)	0.65 (0.59-0.73)	<0.001
Unknown	7,513 (6.0)	145 (1.9)	/	/

^a^ Patients were classified as ICD-O-3 codes “8140-8389: adenomas and adenocarcinomas” and registered as “One primary only” in SEER database. ^b^ Unmarried status included “domestic partner”. Abbreviation: CSS: Cancer-specific Survival; OR: Odds Ratio; 95%CI: 95% confidence interval; Ref: Reference; NH: non-Hispanic; API: Asian or Pacific Islander; AI/AN: American Indian/Alaska Native; RUCA: Rural Urban Commuting Area.

**Table 2:** Univariate cancer-specific survival analysis of primary thyroid cancer patients^a^ in entire cohort and who underwent surgery

Characteristics	Entire Cohort (n = 126,160)		Surgery (n = 122,675)	
	Crude HR_CSS_ (95%CI)	*P*	Crude HR_CSS_ (95%CI)	*P*
**Gender**				
Male	1.00 (Ref.)	/	1.00 (Ref.)	/
Female	0.36 (0.33-0.39)	<0.001	0.36 (0.33-0.39)	<0.001
**Race/Ethnicity**				
NH White	1.00 (Ref.)	/	1.00 (Ref.)	/
NH Black	1.07 (0.91-1.26)	0.40	1.32 (1.14-1.58)	<0.001
NH API	1.14 (1.07-1.21)	<0.001	1.28 (1.20-1.36)	<0.001
NH AI/AN	1.11 (0.92-1.33)	0.28	1.20 (1.00-1.44)	0.052
Hispanic	1.04 (1.01-1.07)	0.004	1.10 (1.07-1.14)	<0.001
NH Unknown	0.67 (0.54-0.84)	<0.001	0.71 (0.57-0.89)	0.003
**Insurance Status**				
Insured	1.00 (Ref.)	/	1.00 (Ref.)	/
Medicaid	2.15 (1.84-2.52)	<0.001	2.99 (2.53-3.50)	<0.001
Uninsured	1.17 (0.99-1.39)	0.07	1.37 (1.15-1.62)	<0.001
**Marital**				
Married	1.00 (Ref.)	/	1.00 (Ref.)	/
Single	0.79 (0.70-0.89)	<0.001	0.95 (0.84-1.07)	0.40
Divorced	1.24 (1.15-1.33)	<0.001	1.35 (1.26-1.46)	<0.001
Widowed	1.81 (1.74-1.87)	<0.001	1.92 (1.85-2.00)	<0.001
Separated	0.97 (0.85-1.10)	0.63	1.02 (0.89-1.15)	0.81
Unmarried	/	/	/	/
**RUCA Category**				
Urban	1.00 (Ref.)	/	1.00 (Ref.)	/
Rural	1.38 (1.20-1.58)	<0.001	1.41 (1.21-1.65)	<0.001
**Yost tertile**				
Low	1.00 (Ref.)	/	1.00 (Ref.)	/
Middle	0.83 (0.75-0.92)	<0.001	1.08 (0.97-1.21)	0.16
High	0.78 (0.74-0.82)	<0.001	0.89 (0.84-0.94)	<0.001
**Stage**				
Localized	1.00 (Ref.)	/	1.00 (Ref.)	/
Regional	5.73 (5.05-6.49)	<0.001	6.24 (5.48-7.10)	<0.001
Distant	9.70 (9.15-10.29)	<0.001	10.13 (9.53-10.76)	<0.001
*In situ*	/	/	/	/

^a^ Patients were classified as ICD-O-3 codes “8140-8389: adenomas and adenocarcinomas” and registered as “One primary only” in SEER database. ^b^ Unmarried status included “domestic partner”. Abbreviation: CSS: Cancer-specific Survival; HR: Hazard Ratio; 95%CI: 95% confidence interval; Ref: Reference; NH: non-Hispanic; API: Asian or Pacific Islander; AI/AN: American Indian/Alaska Native; RUCA: Rural Urban Commuting Area.

**Table 3:** Multivariate cancer-specific survival analysis of primary thyroid cancer patients^ a^ in entire cohort and who underwent surgery

Characteristics	Entire Cohort (n = 126,160)		Surgery (n = 122,675)	
	Adjusted HR_CSS_ (95%CI)	*P*	Adjusted HR_CSS_ (95%CI)	*P*
**Gender**				
Male	1.00 (Ref.)	/	1.00 (Ref.)	/
Female	0.47 (0.42-0.53)	<0.001	0.42 (0.36-0.49)	<0.001
**Race/Ethnicity**				
NH White	1.00 (Ref.)	/	1.00 (Ref.)	/
NH Black	1.00 (0.79-1.28)	0.99	0.96 (0.70-1.33)	0.81
NH API	0.91 (0.76-1.09)	0.30	0.77 (0.60-0.98)	0.03
NH AI/AN	0.80 (0.33-1.93)	0.62	0.89 (0.33-2.39)	0.81
Hispanic	0.76 (0.64-0.90)	0.001	0.81 (0.66-1.00)	0.051
NH Unknown	/	/	/	/
**Insurance Status**				
Insured	1.00 (Ref.)	/	1.00 (Ref.)	/
Medicaid	1.38 (1.17-1.64)	<0.001	1.54 (1.24-1.91)	<0.001
Uninsured	1.03 (0.72-1.47)	0.89	1.11 (0.71-1.74)	<0.001
**Marital**				
Married	1.00 (Ref.)	/	1.00 (Ref.)	/
Single	0.75 (0.63-0.89)	0.001	0.65 (0.52-0.81)	<0.001
Divorced	1.68 (1.37-2.05)	<0.001	1.73 (1.34-2.22)	<0.001
Widowed	3.79 (3.22-4.46)	<0.001	4.01 (3.25-4.95)	<0.001
Separated	0.95 (0.51-1.77)	0.87	0.71 (0.29-1.71)	0.44
Unmarried	/	/	/	/
**RUCA Category**				
Urban	1.00 (Ref.)	/	1.00 (Ref.)	/
Rural	1.04 (0.82-1.33)	0.74	1.04 (0.76-1.41)	0.83
**Yost tertile**				
Low	1.00 (Ref.)	/	1.00 (Ref.)	/
Middle	0.92 (0.79-1.07)	0.25	0.88 (0.72-1.06)	0.18
High	0.76 (0.65-0.89)	<0.001	0.77 (0.63-0.94)	0.01
**Stage**				
Localized	1.00 (Ref.)	/	1.00 (Ref.)	/
Regional	5.64 (4.69-6.77)	<0.001	6.90 (5.46-8.71)	<0.001
Distant	79.12 (66.33-94.37)	<0.001	94.86 (75.43-119.28)	<0.001
*In situ*	/	/	/	/

^a^ Patients were classified as ICD-O-3 codes “8140-8389: adenomas and adenocarcinomas” and registered as “One primary only” in SEER database. ^b^ Unmarried status included “domestic partner”. Abbreviation: CSS: Cancer-specific Survival; HR: Hazard Ratio; 95%CI: 95% confidence interval; Ref: Reference; NH: non-Hispanic; API: Asian or Pacific Islander; AI/AN: American Indian/Alaska Native; RUCA: Rural Urban Commuting Area.

## Abbreviations

SEER: Surveillance, Epidemiology, and End Results Program
ICD-O-3: International Classification of Disease for Oncology, 3rd version
HR: hazard ratio
RUCA: Rural Urban Commuting Area codes
OR: odds ratio
95%CI: 95% confidence interval
CSS: cancer-specific survival
OS: overall survival
