# Supplementary material for: Socioeconomic factors are associated with the prognosis of Thyroid Cancer
Source: J Cancer. 2021 Mar 5;12(9):2507–12. doi: 10.7150/jca.52329 (PMC8040706; doi:10.7150/jca.52329)
Supplement: Supplementary file 1 — Supplementary tables. [file jcav12p2507s1.pdf]

Socioeconomic Factors are Associated with the Prognosis of Thyroid Cancer

Supplementary Materials

Table S1. Comparison of survival outcomes by different factors in patient with primary thyroid cancer.

Table S2. Univariate and multivariate overall survival analysis of primary thyroid cancer patients in entire cohort and who underwent surgery.

Table S3. Univariate and multivariate survival analysis of primary thyroid cancer patients who underwent surgery and radiation (n = 60,062) by different factors.

Table S4. Comparison of 5-year and 10-year survival outcomes by different factors in all patients with thyroid cancer.

Table S5. Multivariate survival analysis of all thyroid cancer patients by different factors.

Abbreviation: OS: Overall Survival; CSS: Cancer-specific Survival; OR: Odds Ratio; HR: Hazard Ratio; 95%CI: 95% confidence interval; Ref: Reference; NH: non-Hispanic; API: Asian or Pacific Islander; AI/AN: American Indian/Alaska Native; RUCA: Rural Urban Commuting Area.

Table S1. Comparison of survival outcomes by different factors in patient with primary thyroid cancer [a].

Characteristics	10-year OS (N of Events = 5,473)			5-year CSS (N of Events = 1,706)			5-year OS (N of Events = 3,663)		
	n of Events (%)	OR (95%CI)	*P*	n of Events (%)	OR (95%CI)	*P*	n of Events (%)	OR (95%CI)	*P*
Gender									
Female	3,289 (3.3)	1.00 (Ref.)	/	964 (1.0)	1.00 (Ref.)	/	2,161 (2.2)	1.00 (Ref.)	/
Male	2,184 (7.8)	2.46 (2.32-2.60)	<0.001	742 (2.7)	2.76 (2.50-3.05)	<0.001	1,502 (5.5)	2.53 (2.37-2.71)	<0.001
Race/Ethnicity									
NH White	3,745 (4.6)	1.00 (Ref.)	/	1,092 (2.3)	1.00 (Ref.)	/	2,461 (3.0)	1.00 (Ref.)	/
NH Black	511 (6.0)	1.34 (1.22-1.47)	<0.001	123 (1.5)	0.62 (0.51-0.74)	<0.001	352 (4.2)	1.40 (1.24-1.57)	<0.001
NH API	481 (3.6)	0.78 (0.71-0.86)	<0.001	205 (1.5)	0.66 (0.56-0.75)	<0.001	322 (2.4)	0.80 (0.71-0.90)	<0.001
NH AI/AN	26 (4.6)	1.00 (0.65-1.48)	1.00	11 (1.9)	1.46 (0.72-2.64)	0.29	19 (3.3)	1.12 (0.67-1.76)	0.73
Hispanic	705 (3.5)	0.75 (0.69-0.82)	<0.001	274 (1.3)	1.01 (0.88-1.16)	0.89	506 (2.5)	0.82 (0.75-0.91)	<0.001
NH Unknown	5 (0.3)	/	/	1 (0.1)	/	/	3 (0.2)	/	/
Insurance Status									
Insured	2138 (2.7)	1.00 (Ref.)	/	738 (0.9)	1.00 (Ref.)	/	1,655 (2.1）	1.00 (Ref.)	/
Medicaid	508 (5.3)	2.03 (1.84-2.25)	<0.001	169 (1.8)	1.92 (1.61-2.28)	<0.001	411 (4.3)	2.12 (1.89-2.36)	<0.001
Uninsured	76 (3.1)	1.16 (0.91-1.46)	0.24	29 (1.2)	1.28 (0.85-1.86)	0.24	56 (2.3)	1.10 (0.82-1.44)	0.53
Unknown	2751 (7.9)	/	/	770 (2.2)	/	/	1541 (4.4)	/	/
Marital Status									
Married	2,679 (3.5)	1.00 (Ref.)	/	842 (1.1)	1.00 (Ref.)	/	1721 (2.2)	1.00 (Ref.)	/
Single	874 (3.2)	0.91 (0.85-0.99)	0.03	256 (0.9)	0.85 (0.74-0.98)	0.03	634 (2.3)	1.04 (0.94-1.14)	0.46
Divorced	504 (6.0)	1.76 (1.59-1.94)	<0.001	142 (1.7)	1.55 (1.28-1.85)	<0.001	320 (3.8)	1.72 (1.52-1.95)	<0.001
Widowed	1,090 (20.2)	7.00 (6.58-7.57)	<0.001	380 (7.0)	6.83 (6.02-7.75)	<0.001	763 (14.1)	7.18 (6.56-7.87)	<0.001
Separated	49 (4.5)	1.32 (0.96-1.76)	0.08	10 (0.9)	0.84 (0.40-1.57)	0.70	35 (3.2)	1.46 (1.01-2.05)	0.04
Unmarried [b]	1 (0.5)	0.13 (0.00-0.72)	0.01	0 (0.0)	/	/	1 (0.5)	0.20 (0.01-1.13)	0.10
Unknown	276 (4.0)	/	/	76 (1.1)	/	/	189 (2.7)	/	/
RUCA Category									
Urban	4,650 (4.1)	1.00 (Ref.)	/	1,468 (1.3)	1.00 (Ref.)	/	3,147 (2.8)	1.00 (Ref.)	/
Rural	528 (6.6)	1.69 (1.53-1.85)	<0.001	148 (1.9)	1.43 (1.20-1.70)	<0.001	325 (4.1)	1.48 (1.31-1.66)	<0.001
Unknown	295 (5.3)	/	/	90 (1.6)	/	/	191 (3.5)	/	/
YOST tertile									
Low	1700 (5.9)	1.00 (Ref.)	/	505 (1.8)	1.00 (Ref.)	/	1,155 (4.0)	1.00 (Ref.)	/
Middle	1,833 (4.6)	0.77 (0.72-0.83)	<0.001	581 (1.5)	0.83 (0.74-0.94)	0.003	1,245 (3.1)	0.77 (0.71-0.84)	<0.001
High	1,586 (3.1)	0.52 (0.48-0.55)	<0.001	510 (1.0)	0.57 (0.50-0.65)	<0.001	1,029 (2.0)	0.50 (0.46-0.54)	<0.001
Unknown	354 (4.7)	/	/	110 (1.5)	/	/	234 (3.11)	/	/

[a] Patients were classified as ICD-O-3 codes "8140-8389: adenomas and adenocarcinomas" and registered as "One primary only" in SEER database.
[b] Unmarried status included "domestic partner".

Table S2. Univariate and multivariate overall survival analysis of primary thyroid cancer patients [a] in entire cohort and who underwent surgery.

Characteristics	Entire Cohort (n = 126,160)				Surgery (n = 122,675)			
	Crude HR_{OS} (95%CI)	*P*	Adjusted HR_{OS} (95%CI)	*P*	Crude HR_{OS} (95%CI)	*P*	Adjusted HR_{OS} (95%CI)	*P*
Gender								
Male	1.00 (Ref.)	/	1.00 (Ref.)	/	1.00 (Ref.)	/	1.00 (Ref.)	/
Female	0.43 (0.41-0.45)	<0.001	0.42 (0.39-0.45)	<0.001	0.43 (0.41-0.46)	<0.001	0.38 (0.35-0.42)	<0.001
Race/Ethnicity								
NH White	1.00 (Ref.)	/	1.00 (Ref.)	/	1.00 (Ref.)	/	1.00 (Ref.)	/
NH Black	1.36 (1.25-1.49)	<0.001	1.06 (0.93-1.20)	0.39	1.55 (1.42-1.70)	<0.001	1.05 (0.89-1.24)	0.56
NH API	0.91 (0.87-0.95)	<0.001	0.70 (0.62-0.80)	<0.001	0.97 (0.93-1.02)	0.26	0.62 (0.53-0.74)	<0.001
NH AI/AN	1.04 (0.92-1.18)	0.51	0.80 (0.47-1.35)	0.39	1.09 (0.97-1.23)	0.16	0.80 (0.43-1.50)	0.49
Hispanic	0.97 (0.95-0.99)	<0.001	0.65 (0.58-0.72)	<0.001	1.00 (0.98-1.02)	0.90	0.65 (0.57-0.75)	<0.001
NH Unknown	0.64 (0.54-0.75)	<0.001	/	/	0.66 (0.56-0.77)	<0.001	/	/
Insurance Status								
Insured	1.00 (Ref.)	/	1.00 (Ref.)	/	1.00 (Ref.)	/	1.00 (Ref.)	/
Medicaid	2.42 (2.20-2.67)	<0.001	1.70 (1.53-1.88)	<0.001	2.94 (2.67-3.25)	<0.001	2.05 (1.81-2.31)	<0.001
Uninsured	1.12 (1.00-1.26)	0.048	1.01 (0.81-1.27)	0.91	1.23 (1.10-1.38)	<0.001	1.13 (0.85-1.49)	0.41
Marital								
Married	1.00 (Ref.)	/	1.00 (Ref.)	/	1.00 (Ref.)	/	1.00 (Ref.)	/
Single	0.96 (0.89-1.03)	0.21	0.84 (0.76-0.93)	<0.001	1.07 (1.00-1.15)	0.06	0.85 (0.76-0.97)	0.01
Divorced	1.34 (1.28-1.40)	<0.001	1.73 (1.54-1.94)	<0.001	1.41 (1.35-1.48)	<0.001	1.72 (1.48-1.99)	<0.001
Widowed	1.87 (1.83-1.92)	<0.001	4.70 (4.26-5.18)	<0.001	1.95 (1.91-2.00)	<0.001	5.44 (4.82-6.15)	<0.001
Separated	1.08 (1.01-1.16)	0.02	1.15 (0.81-1.63)	0.44	1.12 (1.04-1.19)	0.001	1.20 (0.77-1.84)	0.42
Unmarried [b]	0.78 (0.53-1.16)	0.22	0.60 (0.15-2.42)	0.48	0.82 (0.56-1.22)	0.33	0.47 (0.07-3.33)	0.45
RUCA Category								
Urban	1.00 (Ref.)	/	1.00 (Ref.)	/	1.00 (Ref.)	/	1.00 (Ref.)	/
Rural	1.44 (1.33-1.57)	<0.001	1.09 (0.95-1.24)	0.21	1.50 (1.37-1.64)	<0.001	1.06 (0.90-1.25)	0.47
Yost tertile								
Low	1.00 (Ref.)	/	1.00 (Ref.)	/	1.00 (Ref.)	/	1.00 (Ref.)	/
Middle	0.76 (0.71-0.80)	<0.001	0.79 (0.72-0.86)	<0.001	0.87 (0.82-0.93)	<0.001	0.82 (0.73-0.91)	<0.001
High	0.70 (0.68-0.72)	<0.001	0.58 (0.52-0.63)	<0.001	0.75 (0.73-0.78)	<0.001	0.60 (0.53-0.67)	<0.001
Stage								
Localized	1.00 (Ref.)	/	1.00 (Ref.)	/	1.00 (Ref.)	/	1.00 (Ref.)	/
Regional	1.53 (1.44-1.63)	<0.001	1.48 (1.36-1.61)	<0.001	1.62 (1.52-1.72)	<0.001	1.55 (1.40-1.72)	<0.001
Distant	3.93 (3.81-4.05)	<0.001	11.10 (10.15-12.15)	<0.001	4.04 (3.92-4.17)	<0.001	11.64 (10.37-13.07)	<0.001
In situ	0.99 (0.80-1.23)	0.95	/	/	1.00 (0.80-1.24)	1.00	/	/

[a] Patients were classified as ICD-O-3 codes “8140-8389: adenomas and adenocarcinomas” and registered as “One primary only” in SEER database.
[b] Unmarried status included “domestic partner”.

Table S3. Univariate and multivariate survival analysis of primary thyroid cancer patients[a] who underwent surgery and radiation (n = 60,062) by different factors.

Characteristics	Cancer-specific Survival				Overall Survival			
	Crude HR_{CSS} (95%CI)	*P*	Adjusted HR_{CSS} (95%CI)	*P*	Crude HR_{OS} (95%CI)	*P*	Adjusted HR_{OS} (95%CI)	*P*
Gender								
Male	1.00 (Ref.)	/	1.00 (Ref.)	/	1.00 (Ref.)	/	1.00 (Ref.)	/
Female	0.36 (0.32-0.40)	<0.001	0.44 (0.36-0.53)	<0.001	0.41 (0.38-0.44)	<0.001	0.39 (0.35-0.45)	<0.001
Race/Ethnicity								
NH White	1.00 (Ref.)	/	1.00 (Ref.)	/	1.00 (Ref.)	/	1.00 (Ref.)	/
NH Black	1.00 (0.85-1.19)	0.97	0.95 (0.63-1.42)	0.80	1.59 (1.45-1.75)	<0.001	0.85 (0.65-1.12)	0.26
NH API	1.11 (1.03-1.18)	0.003	0.65 (0.48-0.87)	0.005	0.99 (-.94-1.03)	0.54	0.60 (0.48-0.75)	<0.001
NH AI/AN	1.09 (0.91-1.31)	0.37	1.48 (0.47-4.61)	0.503	1.10 (0.97-1.24)	0.12	1.37 (0.61-3.06)	0.45
Hispanic	1.03 (1.00-1.06)	0.09	0.72 (0.55-0.93)	0.01	1.01 (0.99-1.03)	0.55	0.66 (0.54-0.79)	<0.001
NH Unknown	0.67 (0.53-0.84)	<0.001	/	/	0.66 (0.56-0.78)	<0.001	/	/
Insurance Status								
Insured	1.00 (Ref.)	/	1.00 (Ref.)	/	1.00 (Ref.)	/	1.00 (Ref.)	/
Medicaid	2.28 (1.93-2.71)	<0.001	1.56 (1.18-2.07)	0.002	3.02 (2.70-3.37)	<0.001	1.79 (1.48-2.16)	<0.001
Uninsured	1.20 (1.00-1.42)	0.046	1.55 (0.93-2.57)	0.09	1.25 (1.11-1.41)	<0.001	1.14 (0.76-1.70)	0.52
Marital								
Married	1.00 (Ref.)	/	1.00 (Ref.)	/	1.00 (Ref.)	/	1.00 (Ref.)	/
Single	0.68 (0.60-0.77)	<0.001	0.52 (0.39-0.68)	<0.001	1.02 (0.94-1.11)	0.60	0.77 (0.65-0.92)	0.005
Divorced	1.15 (1.06-1.24)	<0.001	1.70 (1.26-2.29)	<0.001	1.38 (1.32-1.45)	<0.001	1.84 (1.50-2.27)	<0.001
Widowed	1.72 (1.65-1.78)	<0.001	3.32 (2.52-4.37)	<0.001	1.92 (1.87-1.97)	<0.001	4.81 (4.00-5.79)	<0.001
Separated	0.93 (0.82-1.06)	0.30	1.04 (0.43-2.53)	0.93	1.10 (1.03-1.18)	0.004	1.28 (0.70-2.33)	0.42
Unmarried[b]	/	/	/	/	0.82 (0.55-1.21)	0.31	0.92 (0.13-6.52)	0.93
RUCA Category								
Urban	1.00 (Ref.)	/	1.00 (Ref.)	/	1.00 (Ref.)	/	1.00 (Ref.)	/
Rural	1.31 (1.08-1.59)	0.007	0.87 (0.57-1.30)	0.49	1.42 (1.25-1.62)	<0.001	1.01 (0.79-1.30)	0.94
Yost tertile								
Low	1.00 (Ref.)	/	1.00 (Ref.)	/	1.00 (Ref.)	/	1.00 (Ref.)	/
Middle	0.84 (0.74-0.96)	0.010	0.90 (0.71-1.15)	0.41	0.95 (0.87-1.04)	0.24	0.83 (0.71-0.98)	0.02
High	0.78 (0.73-0.84)	<0.001	0.76 (0.60-0.98)	0.03	0.79 (0.75-0.82)	<0.001	0.62 (0.52-0.74)	<0.001
Stage								
Localized	1.00 (Ref.)	/	1.00 (Ref.)	/	1.00 (Ref.)	/	1.00 (Ref.)	/
Regional	5.44 (4.59-6.44)	<0.001	5.58 (4.04-7.71)	<0.001	2.05 (1.89-2.22)	<0.001	1.81 (1.56-2.11)	<0.001
Distant	9.30 (8.57-10.08)	<0.001	77.92 (56.75-106.99)	<0.001	4.47 (4.29-4.66)	<0.001	14.64 (12.44-17.24)	<0.001
In situ	/	/	/	/	1.03 (0.83-1.28)	0.79	/	/

[a] Patients were classified as ICD-O-3 codes “8140-8389: adenomas and adenocarcinomas” and registered as “One primary only” in SEER database.
[b] Unmarried status included “domestic partner”.

Table S4. Comparison of 5-year and 10-year survival outcomes by different factors in all patients with thyroid cancer [a].

Characteristics	Thyroid Cancer[a] (N = 136,313)	5-year CSS (Events = 1,919)			5-year OS (Events = 4,679)			10-year CSS (Events = 2,572)			10-year OS (Events = 7,348)		
		N of events (%)	OR (95%CI)	*P*	N of events (%)	OR (95%CI)	*P*	N of events (%)	OR (95%CI)	*P*	N of events (%)	OR (95%CI)	*P*
Gender													
Female	105,508 (77.4)	1082 (1.03)	1.00 (Ref.)	/	2771 (2.63)	1.00 (Ref.)	/	1452 (1.38)	1.00 (Ref.)	/	4449 (4.22)	1.00 (Ref.)	/
Male	30,805 (22.6)	837 (2.72)	2.70 (2.46-2.96)	<0.001	1908 (6.19)	2.45 (2.30-2.60)	<0.001	1120 (3.64)	2.70 (2.50-2.93)	<0.001	2899 (9.41)	2.36 (2.25-2.48)	<0.001
Race/Ethnicity													
NH White	89,388 (65.6)	1242 (1.39)	1.00 (Ref.)	/	3200 (3.58)	1.00 (Ref.)	/	1656 (1.85)	1.00 (Ref.)	/	5121 (5.73)	1.00 (Ref.)	/
NH Black	9,165 (6.7)	139 (1.52)	1.09 (0.91-1.31)	0.35	442 (4.82)	1.36 (1.23-1.51)	<0.001	175 (1.91)	1.03 (0.88-1.21)	0.73	666 (7.27)	1.29 (1.18-1.40)	<0.001
NH API	14,244 (10.4)	225 (1.58)	1.14 (0.98-1.32)	0.08	402 (2.82)	0.78 (0.70-0.87)	<0.001	324 (2.27)	1.23 (1.09-1.39)	<0.001	633 (4.44)	0.77 (0.70-0.83)	<0.001
NH AI/AN	607 (0.4)	11 (1.81)	1.31 (0.65-2.37)	0.48	24 (3.95)	1.11 (0.70-1.67)	0.70	14 (2.31)	1.25 (0.68-2.12)	0.50	33 (5.43)	0.95 (0.64-1.35)	0.82
Hispanic	21,433 (15.7)	301 (1.40)	1.01 (0.89-1.15)	0.89	608 (2.84)	0.79 (0.72-0.86)	<0.001	401 (1.87)	1.01 (0.90-1.13)	0.88	890 (4.15)	0.71 (0.66-0.77)	<0.001
NH Unknown	1,476 (1.1)	1 (0.07)	0.05 (0.00-0.27)	<0.001	3 (0.20)	0.05 (0.01-0.16)	<0.001	2 (0.14)	0.07 (0.01-0.26)	<0.001	5 (0.34)	0.06 (0.02-0.13)	<0.001
Insurance Status													
Insured	84,228 (61.8)	841 (1.00)	1.00 (Ref.)	/	2193 (2.60)	1.00 (Ref.)	/	1009 (1.20)	1.00 (Ref.)	/	2917 (3.46)	1.00 (Ref.)	/
Medicaid	10,008 (7.3)	188 (1.88)	1.90 (1.61-2.23)	<0.001	480 (4.80)	1.88 (1.70-2.09)	<0.001	216 (2.16)	1.82 (1.56-2.11)	<0.001	605 (6.05)	1.79 (1.64-1.96)	<0.001
Uninsured	2,559 (1.9)	33 (1.29)	1.30 (0.88-1.84)	0.18	70 (2.74)	1.05 (0.81-1.34)	0.73	38 (1.48)	1.24 (0.87-1.72)	0.22	95 (3.71)	1.07 (0.86-1.32)	0.53
Unknown	39,518 (29.0)	857 (2.17)	/	/	1936 (4.90)	/	/	1309 (3.31)	/	/	3731 (9.44)	/	/
Marital Status													
Married	83,265 (61.1)	960 (1.15)	1.00 (Ref.)	/	1178 (1.41)	1.00 (Ref.)	/	1360 (1.63)	1.00 (Ref.)	/	3772 (4.53)	1.00 (Ref.)	/
Single	28,796 (21.1)	285 (0.99)	0.86 (0.75-0.98)	0.03	760 (2.64)	1.89 (1.72-2.07)	<0.001	361 (1.25)	0.76 (0.68-0.86)	<0.001	1095 (3.80)	0.83 (0.78-0.89)	<0.001
Divorced	9,249 (6.8)	162 (1.75)	1.53 (1.28-1.81)	<0.001	426 (4.61)	3.36 (3.00-3.77)	<0.001	220 (2.38)	1.47 (1.26-1.70)	<0.001	686 (7.42)	1.69 (1.55-1.84)	<0.001
Widowed	6,177 (4.5)	412 (6.67)	6.13 (5.43-6.91)	<0.001	934 (15.12)	12.41 (11.33-13.60)	<0.001	501 (8.11)	5.32 (4.77-5.91)	<0.001	1384 (22.41)	6.09 (5.68-6.52)	<0.001
Separated	1,170 (0.9)	14 (1.20)	1.04 (0.56-1.76)	1.00	46 (3.93)	2.85 (2.06-3.85)	<0.001	18 (1.54)	0.94 (0.55-1.50)	0.89	66 (5.64)	1.26 (0.97-1.62)	0.08
Unmarried [b]	225 (0.2)	0 (0)	/	/	2 (0.89)	0.62 (0.08-2.29)	0.78	0 (0)	/	/	2 (0.89)	0.19 (0.02-0.69)	0.004
Unknown	7,431 (5.5)	86 (1.16)	/	/	233 (3.14)	/	/	112 (1.51)	/	/	343 (4.62)	/	/
RUCA Category													
Urban	121,526 (89.2)	1651 (1.36)	1.00 (Ref.)	/	4010 (3.30)	1.00 (Ref.)	/	2205 (1.81)	1.00 (Ref.)	/	6231 (5.13)	1.00 (Ref.)	/
Rural	8,783 (6.4)	165 (1.88)	1.39 (1.18-1.63)	<0.001	426 (4.85)	1.49 (1.35-1.66)	<0.001	230 (2.62)	1.46 (1.26-1.67)	<0.001	714 (8.13)	1.64 (1.51-1.78)	<0.001
Unknown	6,004 (4.4)	103 (1.72)	/	/	243 (4.05)	/	/	137 (2.28)	/	/	403 (6.71)	/	/
Yost tertile													
Low	30,991 (22.7)	555 (1.79)	1.00 (Ref.)	<0.001	1462 (4.72)	1.00 (Ref.)	<0.001	711 (2.29)	1.00 (Ref.)	<0.001	2254 (7.27)	1.00 (Ref.)	<0.001
Middle	42,613 (31.3)	654 (1.53)	0.85 (0.76-0.96)	0.008	1573 (3.69)	0.77 (0.72-0.83)	<0.001	855 (2.01)	0.87 (0.79-0.97)	0.008	2403 (5.64)	0.76 (0.72-0.81)	<0.001
High	54,583 (40.0)	586 (1.07)	0.60 (0.53-0.67)	<0.001	1351 (2.48)	0.51 (0.48-0.55)	<0.001	843 (1.54)	0.67 (0.60-0.74)	<0.001	2209 (4.05)	0.54 (0.51-0.57)	<0.001
Unknown	8,126 (6.0)	124 (1.53)	/	/	293 (3.61)	/	/	163 (2.01)	/	/	482 (5.93)	/	/

[a] Patients were classified as ICD-O-3 codes “8140-8389: adenomas and adenocarcinomas”.
[b] Unmarried status included “domestic partner”.

Table S5. Multivariate survival analysis of all thyroid cancer patients [a] by different factors

Characteristics	Cancer-specific Survival		Overall Survival	
	Adjusted HR_{CSS} (95%CI)	*P*	Adjusted HR_{OS} (95%CI)	*P*
Gender				
Male	Ref.	/	Ref.	/
Female	0. 38 (0.35-0.41)	<0.001	0.45 (0.43-0.47)	<0.001
Race/Ethnicity				
NH White	Ref.	/	Ref.	/
NH Black	1.11 (0.96-1.29)	0.18	1.33 (1.23-1.43)	<0.001
NH API	1.15 (1.08-1.21)	<0.001	0.92 (0.88-0.95)	<0.001
NH AI/AN	1.12 (0.94-1.33)	0.19	1.04 (0.93-1.15)	0.50
Hispanic	1.05 (1.02-1.08)	<0.001	0.96 (0.94-0.97)	<0.001
NH Unknown	0.67 (0.53-0.83)	<0.001	0.61 (0.52-0.72)	<0.001
Insurance Status				
Insured	Ref.	/	Ref.	/
Medicaid	2.12 (1.83-2.45)	<0.001	2.16 (1.98-2.35)	<0.001
Uninsured	1.16 (0.99-1.36)	0.07	1.09 (0.98-1.21)	0.10
Marital Status				
Married	Ref.	/	Ref.	/
Single	0.79 (0.71-0.89)	<0.001	0.88 (0.82-0.93)	<0.001
Divorced	1.23 (1.15-1.31)	<0.001	1.30 (1.25-1.35)	<0.001
Widowed	1.74 (1.69-1.80)	<0.001	1.77 (1.74-1.80)	<0.001
Separated	1.00 (0.90-1.12)	0.96	1.08 (1.02-1.14)	0.01
Unmarried [b]	/	/	0.86 (0.66-1.14)	0.31
RUCA Category				
Urban	Ref.	/	Ref.	/
Rural	1.31 (1.15-1.49)	<0.001	1.44 (1.34-1.54)	<0.001
Yost tertile				
Low	Ref.	/	Ref.	/
Middle	0.83 (0.76-0.92)	<0.001	0.75 (0.71-0.79)	<0.001
High	0.78 (0.75-0.82)	<0.001	0.72 (0.70-0.73)	<0.001

[a] Patients were classified as ICD-O-3 codes “8140-8389: adenomas and adenocarcinomas”.
[b] Unmarried status included “domestic partner”.
